# Feasibility study of a home-based sensory training system (STS) device for type 1 complex regional pain syndrome in England: Lessons learnt

**DOI:** 10.1177/20494637251371592

**Published:** 2025-08-29

**Authors:** Jessica Coggins, Sharon Grieve, Lisa Buckle, Darren Hart, Alison Llewellyn, Mark Palmer, Moniek Wittens, Candida McCabe

**Affiliations:** 1 School of Health and Social Wellbeing, 1981 University of the West of England, Bristol, UK; 2 Research and Development Department, 1556 Royal United Hospitals Bath NHS Foundation Trust, Bath, UK; 3 Medical Physics & Bioengineering Department, 1556 Royal United Hospitals Bath NHS Foundation Trust, Bath, UK; 4 Department of Computer Science and Creative Technologies, 1981 University of the West of England, Bristol, UK; 5 PPI Contributor, School of Health and Social Wellbeing, 1981 University of the West of England, Bristol, UK

**Keywords:** complex regional pain syndrome, sensory training system, home use, sensory discrimination, wearable, device, electrical stimulation, game-based therapy

## Abstract

**Introduction:**

Sensory discrimination training has demonstrated improvements in two-point discrimination and pain reduction in people with chronic pain. We tested the feasibility and acceptability of a novel Sensory Training System (STS) device in the homes of people with Type 1 Complex Regional Pain Syndrome (CRPS).

**Methods:**

Participants meeting CRPS diagnostic criteria were invited to use the STS for a minimum of 30 minutes per day for 30 days. Device usage data were captured by the STS. Assessments at baseline and after 30 days were: two-point discrimination ability, pain intensity and interference, sensitivity and emotions towards CRPS limb. Qualitative interviews were conducted at the end of the study to capture participants’ feedback on the device.

**Results:**

A total of 10 participants (female n = 7) completed the study. Participants’ mean age was 56.4 years (range: 24–78 years), and mean disease duration was 9.37 years (range: 4.25–26.5). Eight had lower limb CRPS. The mean STS device use was 27.3 ± 3.4 days and mean daily usage of training games was 00:27:11 ± 00:07:52 (hh:mm:ss). No patterns or trends were evident between device usage and outcome data.

**Conclusion:**

This feasibility study of a home-based STS for people with CRPS revealed key areas for improvement in the device’s hardware and software and outlined the challenges of development and testing during the COVID-19 pandemic, while also capturing valuable usability insights from participant feedback. Key recommendations include early and ongoing collaboration with users, securing sufficient funding, ensuring correct device setup by participants, conducting interim analysis, and using online tools to enhance participant experience and data collection.

**Study registration:**

The study was registered with ISRCTN registry on 28^th^ May 2021 (https://doi.org/10.1186/ISRCTN89099843).

## Introduction

Chronic pain affects between one third and one half of the UK population, and is associated with reduced tactile acuity in neuropathic and non-neuropathic pain groups.^1–7^ Tactile acuity, assessed using two-point discrimination (TPD), is the ability to define two tactile stimuli when simultaneously placed on the skin, and is thought to provide an insight into somatosensory cortical functioning.^8,9^ The shorter the distance between the two perceived stimuli, the better the tactile acuity, and the better defined that area is on the somatosensory cortex.^9^ Poor TPD is associated with higher intensity of pain experienced^2–6,10^ and with altered brain representation of the affected body part.^5,6^

In Type 1 Complex Regional Pain Syndrome (CRPS), a chronic pain condition of the limbs that commonly occurs after trauma,^11,12^ individuals frequently lose the ability to determine texture, temperature, and location of stimulus on their painful skin.^7^ Furthermore, non-painful stimuli, for example, light touch, are perceived as painful (allodynia).^13–16^ The personal impact of these altered perceptions can be life changing for those living with CRPS; and often results in lower quality of life,^17^ hostile feelings towards their affected limb,^15^ and an intolerance of clothing on the painful area which can lead to fear of touch and social isolation.^15,16^

Interventions to improve TPD and reduce pain by normalising brain representation have been developed and tested.^18–34^ These interventions seek to reverse pain-related cortical adaptations in sensory perception by helping the individual to broaden their sensory experience, away from just perceiving pain, thereby ‘re-finding’ normal sensations and relieving chronic pain. Previous studies in chronic pain populations have demonstrated sensory discrimination training involving cognitive effort, can improve TPD, reduce patient-reported pain, and normalise the cortical representation of the painful area.^18,21^

Despite the research, sensory discrimination training in clinical practice remains sub-optimal. It often involves the manual application of stimuli by a therapist or carer, which is slow to deliver improvements and has poor adherence.^26^ Since 2008 and inspired by Flor and colleagues,^16^ our research group has been developing a Sensory Training System (STS) for home use to improve sensory discrimination for people with CRPS. Starting with healthy volunteers^35^ and cumbersome hardware and software that required a technician to operate, we progressed into clinical studies with CRPS patients participating in a residential functional rehabilitation programme.^31^ For this first study with patients we had progressed the software programme to provide a range of challenges, but the system required manual operation by a technician. With further developments to our hardware and software, we were able to progress to a device that could be independently used by CRPS patients in their homes.^32^ Previous studies of home-based device use have demonstrated the importance of having a well-developed, reliable device, which is suitable for unsupervised independent use if optimal compliance and envisaged benefit are to be gained. With this in mind, we have worked closely with people with CRPS to help determine hardware and software system requirements and demonstrated our device can improve sensory discrimination in healthy volunteers and people with CRPS.^31,32,35^

**Figure 1.**
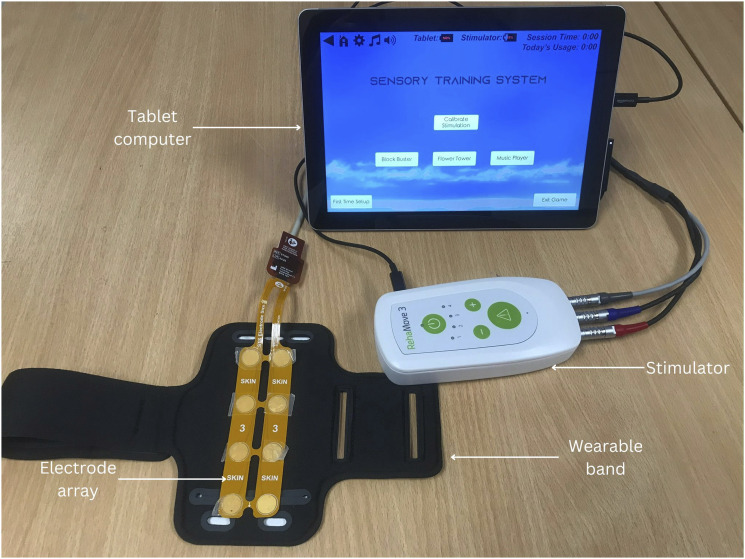
STS device.

Our STS comprises a wearable, and tablet computer (Figure 1), delivering a non-painful stimulation in a random order to four electrode pairs (electrode array) on the wearable. The participant makes a judgement, via the tablet computer touchscreen, about which electrodes have been stimulated. Software programmes deliver training via engaging ‘games’ and task difficulty can be adjusted.

A therapeutic ‘dose’ of sensory training, shown to improve TPD and reduce pain in amputee phantom limb pain, has been proposed as between 30 and 60 min per day over a 30-day period.^18^ Using this evidence, we sought to evaluate the feasibility and practicality of people with CRPS using our STS at home for this duration. We also wished to assess participant engagement and ease of use.

We present the findings of our STS feasibility study and discuss recommendations for future device trials that seek to improve sensory discrimination for people with CRPS for the relief of chronic pain.

## Methods

### Study design

This single centre, proof-of-concept study was conducted in participants’ homes in England, supported by online or telephone communications from the research team, due to the COVID-19 pandemic. Adhering to UK Government policies on social distancing and infection control, the study spanned from April 2019 to June 2023. Consequently, no face-to-face meetings with patients occurred, except for two participants after restrictions were lifted. The study protocol was registered on the ISRCTN registry on 28^th^ May 2021 (ISRCTN89099843) and published.^36^ There was one deviation from the protocol: two home visits to assist with device setup once COVID-19 restrictions were eased. This deviation was discussed with the ethics committee, and appropriate documentation and risk assessments were implemented.

Medicines and Healthcare products Regulatory Agency (MHRA) approval was obtained for the clinical investigation of a non-UKCA/CE UKNI/CE-marked medical device (CI/2021/0019/GB). The study was approved by the London-Stanmore Research Ethics Committee (ref 21/LO/0200) and the Health Research Authority.

### Participants

We aimed to recruit a convenience sample of 20 adults with CRPS, who met the inclusion and exclusion criteria (Table 1), and were willing to use the STS at home for 30 minutes to 2 hours daily over 30-days. The scope of the funding award was sufficient for the development of two pilot STS devices. Including the turnaround time of postage, video calls, setting up the device and preparing the device between participants, this amounted to approximately 45 days per participant, and only two participants could be in the study at any time. As a result, the sample size was pragmatically limited to 20 participants who could complete the study during the funded period.

**Table 1.** Inclusion/ exclusion criteria.

**Inclusion criteria**
• Adults (18+) with upper or lower limb CRPS type I, meeting the Budapest clinical criteria^13^
• An area on their limb where a wearable band with electrode array could be attached near their painful site
• An average pain score in the 7 days prior to baseline data collection, rated as ≥5 by the participant at rest on the numeric rating scale where zero = ‘no pain’ and 10 = ‘the worst pain imaginable’^38^
• Access, and willingness to use appropriate technology to enable full study participation (a smart phone, tablet computer or computer compatible with using the video call function on Microsoft Teams; internet access; an email address; and self-determined physical and mental capacity to tolerate use of technology)
• A friend or family member available to conduct the two-point discrimination measurement
**Exclusion criteria**
• Diagnosis of any other neurological, motor disorder or major nerve damage (including CRPS type II)
• Any mental health condition which may detrimentally impede study participation, in the judgement of the patient or researcher
• Reported presence of any other limb pathology or pain on the affected CRPS limb
• Reported poor skin condition on the area to be stimulated
• Reported poorly controlled epilepsy
• Receipt of intensive CRPS specific multidisciplinary team rehabilitation in an inpatient setting either during the individual’s participation in the study or within the previous month
• Unable to understand written or verbal English and give informed consent
• Reported active medical implants such as cardiac pacemakers or other devices
• Reported exposed orthopaedic metal work in the area of electrical stimulation
• Reported pregnancy
• Known allergy to acrylates which are present in some device components
• Those living alone and not in a COVID-19 ‘support bubble’ with another household *(this applied only when UK government social distancing measures were in place, and was necessary to ensure someone was available to record the TPD measurement)*

Participants were recruited from the CRPS Network UK Registry (https://www.crps.org.uk) or a ‘consent to contact’ list held by the Royal United Hospitals Bath NHS Foundation Trust, UK and overseen by the CRPS Network UK Registry Administrator. The Administrator circulated a study invitation letter and Participant Information Sheet (PIS) to potential participants via email. Interested individuals’ contact details were passed to the research team. After eligibility was confirmed by telephone call with the researcher, participants were emailed an e-consent link on the Qualtrics platform (https://www.qualtrics.com/).^37^ Upon receipt of e-consent, the STS was couriered to their home. The researcher hosted a video call with participants using their own personal devices to answer any questions and guide participants through two training films on a private YouTube channel: (1) conducting the TPD assessment, and (2) how to set up the STS and start the game training. The videos were accompanied by instructions for use booklets, for those who preferred written guidance. Information included potential risks and disadvantages of taking part and when to pause device usage. Participants were able to contact the study team at any point during their participation for support.

The device was collected and returned by courier at the study’s cost when no longer required.

### The sensory discrimination system (STS)

The STS device (Figure 1) consists of four electrode pairs (electrode array), in a flexible wearable band, positioned near, but not on, the allodynia-affected area. Participants guided the wearable’s position to ensure it was placed as close to their painful area as possible whilst remaining comfortable and without exacerbating pain.

Five electrode array sizes were provided, with the smallest being the most challenging due to closer electrode spacing. Participants chose the array sizing with researcher support, based on preference and practicalities of anatomy. Participants could change the array size, guided by the feedback from the device. A 50%–70% training accuracy indicated the correct array size. If accuracy exceeded 70% consistently, participants were prompted to switch to a smaller array or a more challenging level.

The electrode array connected to a stimulator and a tablet computer running the ‘STS software’. The software’s home screen featured a ‘First Time Setup’ menu to set a baseline stimulation current and frequency for all electrodes, ensuring a comfortable but clear sensation. A ‘Calibration Stimulation’ menu, accessed at the start of each session, allowed participants to adjust the stimulation intensity (pulse width) from a default 50% value for each electrode pair, ensuring even sensation across all pairs. This calibration accommodated session-to-session variations in perceived intensity, electrode positioning and contact such that the sensory challenge would be predominantly spatial, as opposed to intensity, discrimination. This menu was accessible throughout use for adjustments. Stimulation intensity ranged from 0% (50µs) to 100% (350µs), with current from 1 to 50 mA. Frequency options were 10 Hz (slow), 25 Hz (medium), or 40 Hz (fast). Spatial stimulation burst duration varied from 900 to 925 ms depending on frequency chosen.

### Game-based training

The tablet computer hosted two bespoke training games, ‘Blockbuster’ and ‘Flower Tower,’ designed to be easy to use and engaging. Participants could select which training game they wished to use and could change games at any time. An additional optional relaxation activity, Music Player, was available. This was suggested by our patient and public involvement (PPI) partners. It allowed participants to listen to music and feel stimulation through the electrodes after 15-minutes of training game play. In Music Player mode, participants did not have to actively engage with the system. The sensation of stimulation was delivered in line with the rhythm of the music. Participants could also visually see the rhythm on the four-bar graphic equaliser displayed on the tablet computer.

**Figure 2.**
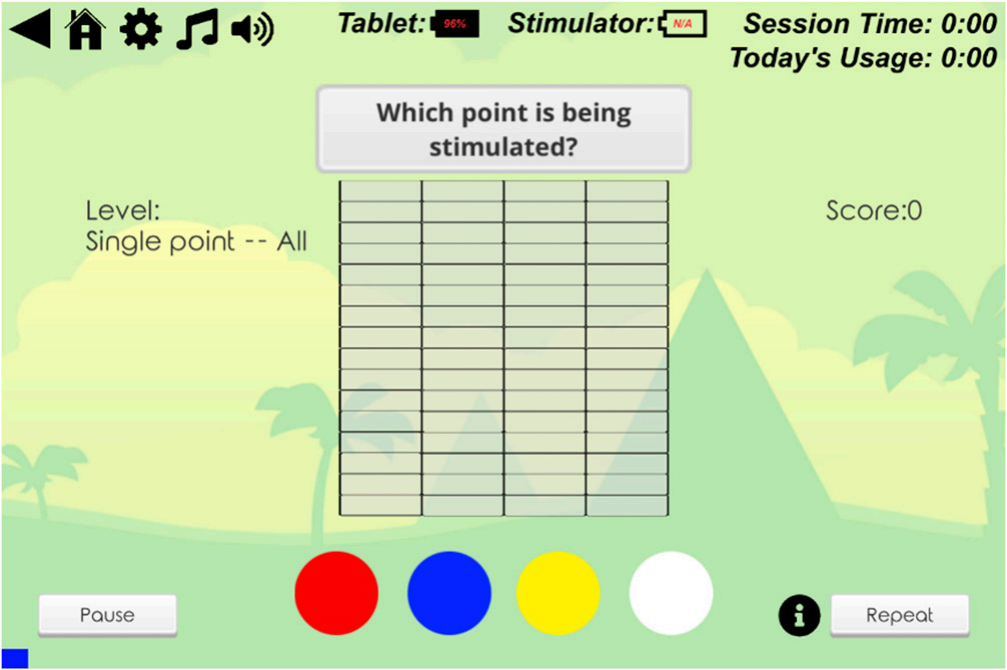
STS Blockbuster single point all level.

**Figure 3.**
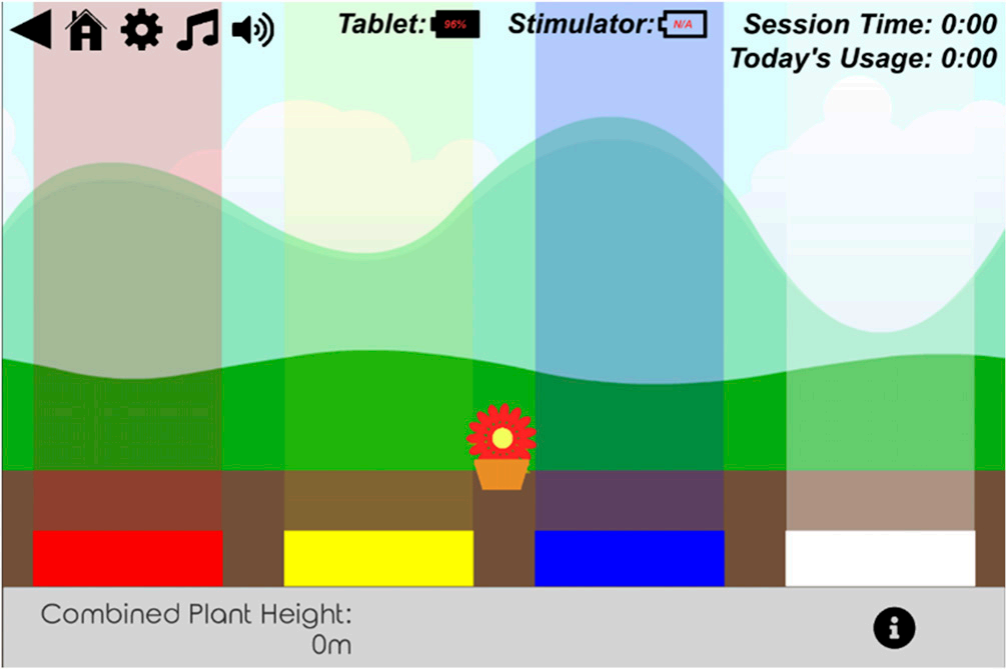
STS flower tower.

‘Blockbuster’ comprised five progressively challenging levels designed to meet varying levels of attainment by participants. In each level, participants identified which of the four electrodes were being stimulated by selecting the corresponding colour on the touch screen (Figure 2). Stimulation could involve single or multiple electrode pairs, with increasing complexity. Participants could repeat the stimulation bursts if required. Encouraging messages, aimed to provide motivation, were displayed on the tablet computer during gameplay. ‘Flower Tower’ was a single level challenge where accurate responses grew a digital flower which could be planted in the greenery when fully grown (Figure 3). Incorrect responses decreased the flower’s height.

The device recorded total daily use (time device turned on and off), and frequency and duration of each game and Music Player session. These data were used to investigate the primary aim of whether participants used the STS for the therapeutic dose of 30 minutes a day for 30 days.

### Data collection

#### Time 1 (T1)

After informed consent, and before the planned video call with the researcher, participants completed a baseline questionnaire via Qualtrics. The questionnaire comprised demographic data, rating of pain intensity, pain interference, sensitivity and emotional feelings of the CRPS limb (see Table 2). Clinical data were collected to detect any trends that emerged and to assess the acceptability and feasibility of the outcome measures employed. The sensory discrimination assessment had to be adapted to enable a family member or friend to conduct this, due to COVID-19 restrictions in place at the time of the study.

**Table 2.** Questionnaire data.

	Data	Description
1	Demographic data	Date of birth, gender, CRPS affected limb(s), CRPS duration, and current medications
2	Assessment of pain intensity	A self-report rating of average pain intensity over the previous 7 days on an 11-point numerical rating scale (0–10) from patient reported outcomes measurement information system (PROMIS®) numeric rating scale v.1.0 -pain intensity 1a.35
3	Assessment of pain interference	A self-report rating of pain interference over the previous 7 days on an 11-point numerical rating scale (0–10). This comprised four items in the PROMIS item bank v1.1 – Pain interference – Short form 4a^39^
4	Assessment of sensitivity	A self-report rating of average sensitivity in the CRPS affected limb over the previous 7 days on an 11-point numerical rating scale (0–10). This aimed to capture how sensitive the participant was to stimuli, such as clothing, on, or close to the affected area
5	Assessment of emotional feelings towards the affected limb	Assessment of emotional feelings towards the affected limb. A self-report rating ranging from strongly positive to strongly negative on an 11-point numerical rating scale (0–10). This was informed by the bath CRPS body perception disturbance scale^40^

### Sensory discrimination assessment

**Figure 4.**
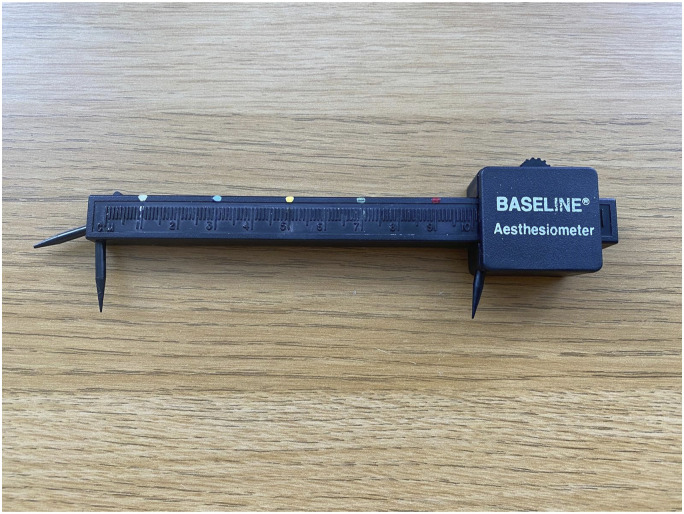
The marked two-point discrimination tool.

Participants identified a friend or family member to conduct this measurement, and they were provided with a short film and information booklet. The participant positioned themselves comfortably, with the test area exposed and eyes closed. A plastic two-point aesthesiometer was used near the area of allodynia. An aesthesiometer is a device used to measure tactile sensitivity. It features two adjustable points, allowing for assessment of the minimal distance between two points that an individual can distinguish. The anatomical location was recorded for repeat testing at Time 2. To simplify the task and to reduce potential measurement errors, each tool had five different coloured marks, spaced 2 cms apart, which indicated when the aesthesiometer was at 1 cm, 3 cm, 5 cm, 7 cm and 9 cm (Figure 4). The person conducting the measurement started at 9 cm and reduced this sequentially according to whether the participant could accurately discriminate between one and two points against their skin. This pragmatic approach was in response to changes required by the COVID-19 pandemic.

Based on prior work,^9^ the sensory discrimination score was the smallest distance at which the participant correctly identified as two points of contact on three occasions. This assessment was completed at the participant’s earliest convenience after the video call.

### Participant diary

Participants received a 30-day paper diary to record array size changes and document their STS use experiences. Completed diaries were returned to the research team to identify device-related issues. Any issues raised by a participant, were discussed in more detail in their qualitative interviews.

#### Time 2 (T2)

Assessments were repeated after 30 days of STS use and recorded on a T2 questionnaire via Qualtrics. Four additional questions to T1 assessed perceptions of change of pain intensity, pain interference, sensitivity, and emotional feelings towards the affected limb rated on a 7-point Likert scale ranging from ‘very much improved’ to ‘very much worse’. The sensory discrimination test was repeated, and outcome recorded in the questionnaire.

### Qualitative data collection

Usability and acceptability of STS use was captured via semi-structured telephone interviews within 14 days of completing device use to support the mitigation of recall bias. Open questions explored device usability and acceptability. With consent, interviews were recorded, transcribed, checked for accuracy, and anonymised.

### Device data

Device data included usage dates, time spent on each activity, and scores. The STS software exported these data into a Comma Separated Values (CSV) format.

### Data analysis

Questionnaire data were transferred from Qualtrics into Microsoft Excel and analysed using descriptive statistics. As a feasibility study, and not a definitive trial, data were examined for patterns in use of the STS, changes in participants’ sensory discrimination, perceived pain intensity, pain interference, sensitivity, emotional feelings about their affected limb, and self-report questionnaire data. PROMIS interference items were scored using either the online ‘Assessment Center Scoring Service’ for assessment of fully anonymised data or by referring to the raw score look-up table in the PROMIS scoring manual (https://www.healthmeasures.net/).

Interview data were thematically analysed^41^ to identify any commonalities about the usability and practicality of device use, from participants’ perspectives. Interview transcripts were read and re-read to gain an understanding and familiarisation with users’ experiences. Following the qualitative descriptive approach,^42^ descriptive labels (codes) were identified and grouped together to form themes. These were reviewed and further defined before being written up for reporting.

Device data were transferred from the tablet computers to a secure shared drive using a USB memory stick and stored individually before copies were consolidated into a Microsoft Excel spreadsheet. Data were arranged in a standardised format to enable analysis and identification of missing data, prior to the conduct of descriptive analysis.

Data extracted from the tablets computer’s operating systems log was managed in a similar manner, when encountering data loss issues for some participants.

### Patient and public involvement and engagement

Patient involvement was crucial in shaping this study. We have worked alongside and consulted with people with CRPS since 2010, when we were in the early stages of device development. People with CRPS informed the design and functioning of the STS hardware and software for the MHRA submission and study conduct. A public contributor, part of the project management group, participated in all monthly meetings, helped design patient-related documentation, and reviewed dissemination reports, publications, and summaries.

### Adverse event recording

Adverse events were considered as any unwanted effects detected by participants during the study, regardless of their attribution to the STS. Participants were instructed during the video calls and in written instructions to contact the study team to report such concerns promptly. All adverse events were reported to the Chief Investigator and documented using a pre-determined form. The research team discussed appropriate responses, often advising participants to cease using the STS device. In the event of a serious adverse event, serious adverse reaction, or suspected unexpected serious adverse reaction, the research team would immediately inform the CI, sponsor, and MHRA. A quarterly report of these events was provided to the MHRA.

## Results

### Participants

**Figure 5.**
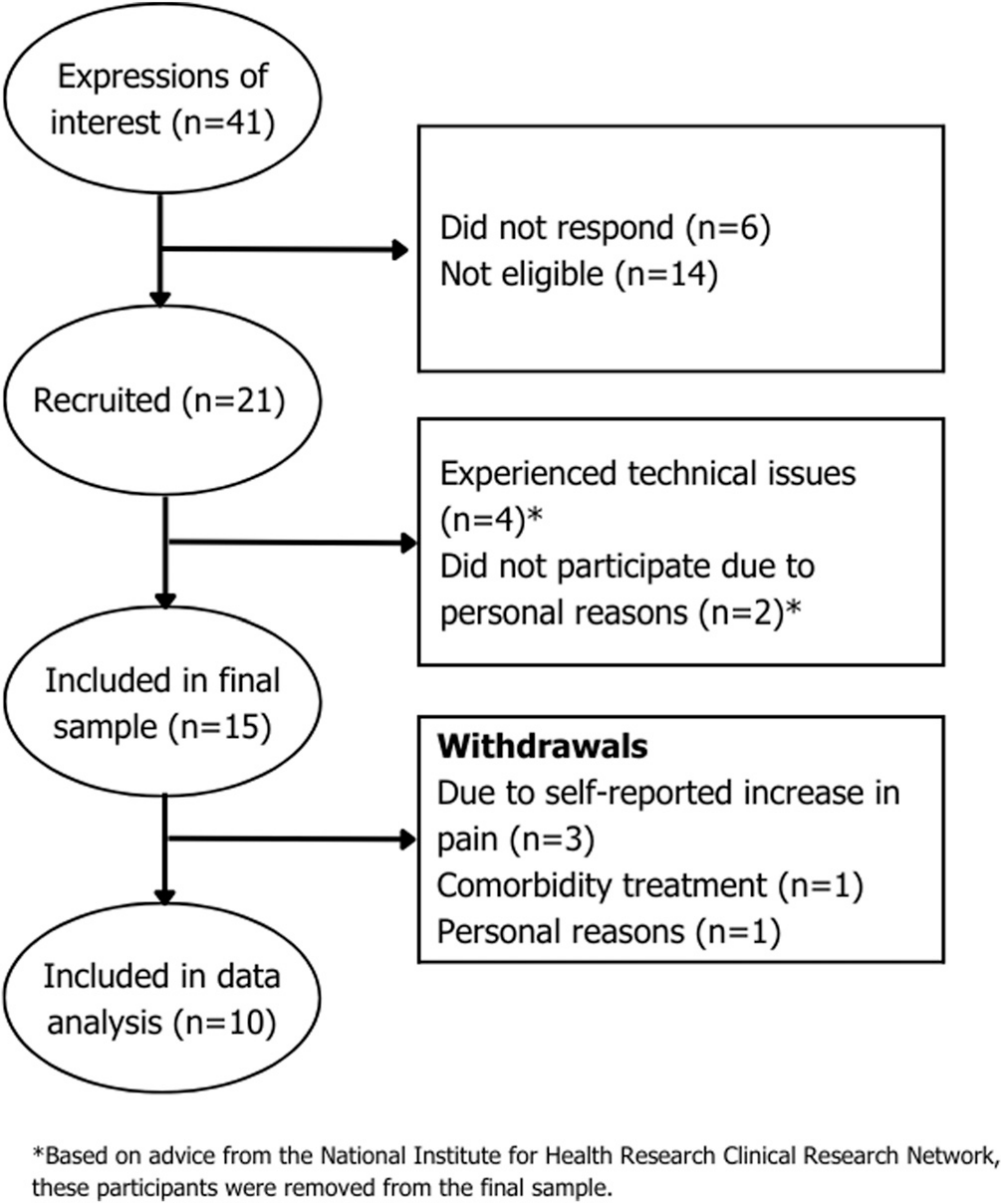
Participant recruitment summary.

Forty-one individuals expressed an interest in participating, 21 participants were recruited between September 2021 and April 2023, and 15 participants were included in the final sample. Please see Figure 5 for further information. Five participants withdrew and data analysis was performed on the remaining 10 participants, with a mean age of 56.4 years (range: 24 to 78 years; median: 61.5). The mean disease duration was 9.37 years (range: 4.25 to 26.5 years; median: 8.3), and there were seven females and three males among the participants. CRPS affected six participants in their right leg/foot, one in their right arm/hand, two in their left leg/foot, and one in their left arm/hand.

## Quantitative findings

### Duration of STS use

Participants used the STS for an average of 27.3 ± 3.4 days. They engaged with the training games for a minimum of 30 minutes on an average of 14 ± 10.7 days this extended to 22.5 ± 6.4 days when including the Music Player. Four participants used the device daily for 30 days, but none used the STS for 30-minutes a day for 30 consecutive days.

The mean daily usage for the training games was 00:27:11 ± 00:07:52 (hh:mm:ss). Including Music Player, daily usage extended to 00:30:48 ± 00:06:42 (hh:mm:ss). The mean *total* usage over 30 days for the games was 13:35:44 ± 03:56:03 (hh:mm:ss), and 15:19:50 ± 03:26:59 (hh:mm:ss) when including Music Player. There was no discernible pattern between participant age and total hours of device use, or between total use and CRPS-affected limb.

### Device data completeness

Device usage data showed a 13% missing rate due to a software issue discovered at study’s end. As it was unforeseen, there was no prior plan to manage this type of missing data. Data were saved only when the ‘exit game’ button was used, not when the device was shutdown using the power button. Consequently, one participant had no device usage data for 30 days, and their self-reported diary entries were used instead, matching startup and shutdown times from the tablet computer’s operating systems log (Window Event Viewer). Four participants had device usage data missing for one to three days. A mean of daily device use was calculated from their remaining data and applied to days where device usage was evident from participant diaries or the tablet computer’s operating systems log. There were no days without any recorded device data, daily diaries or evidence of device use.

### Self-reported questionnaire data

All participants included in the analysis completed questionnaires 1 and 2 fully, meaning that the two-point discrimination data, pain interference, pain intensity, sensitivity and feelings towards the limb data was complete.

### Two-point discrimination data

Four participants improved by one or more points in TPD (one point equates to 2 cm) between T1 and T2. Three participants exhibited no discernible change, and three others experienced a decline. Descriptive analysis of data did not reveal any patterns between TPD outcomes and device usage, diagnosis duration or affected limb.

### Pain interference and intensity

Pain interference for all participants at T1 was 1 standard deviation (SD) worse than the PROMIS^35^ general population. No meaningful change was observed between T1 and T2. However, three participants did withdraw from the study due to exacerbated symptoms.

### Sensitivity and feelings towards the limb

Five participants reported no change in perceived sensitivity, three reported improvements, and two reported increased sensitivity. Regarding feelings towards the limb, one participant noted an improvement, four reported no change and five reported a deterioration.

**Figure 6.**
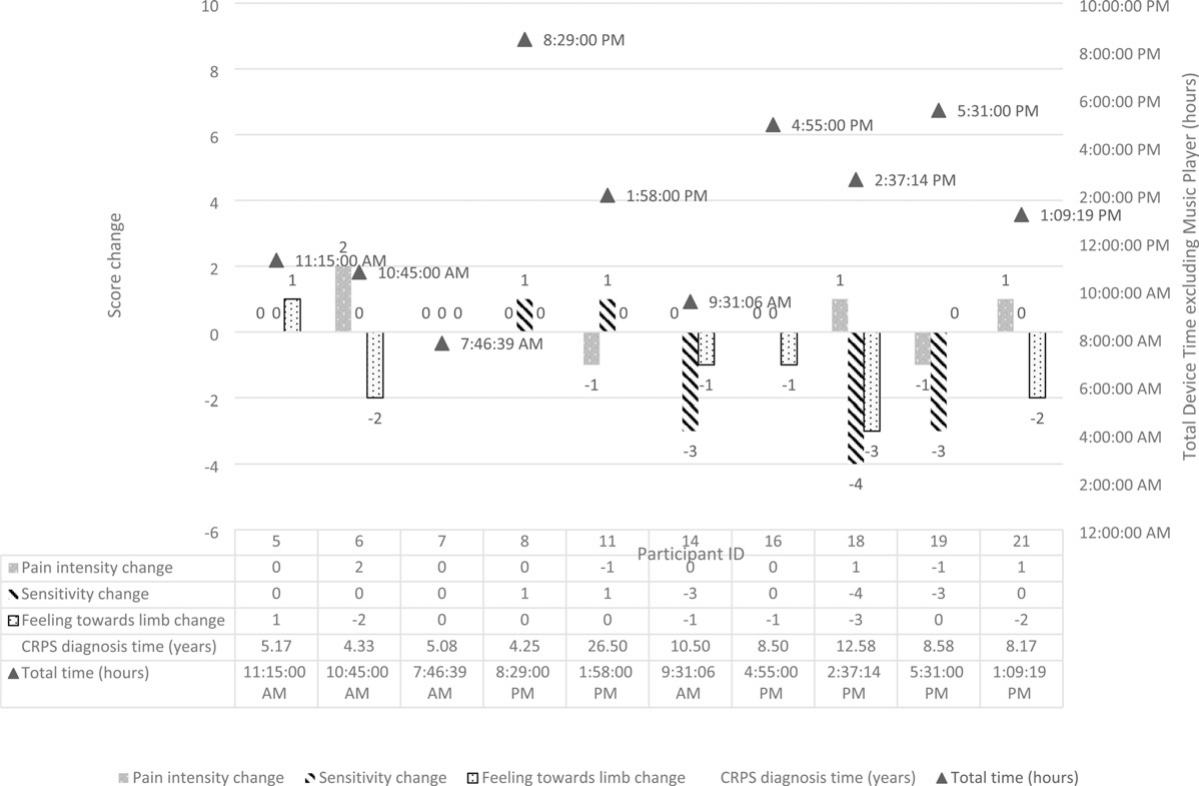
Changes in pain intensity, sensitivity, and feelings towards the limb scores (left sided values), and device use time excluding Music Player (right hand values) in individual participants.

No discernible pattern emerged when comparing pain interference, pain intensity, sensitivity, and feelings towards the limb with total time and days used of the device, or diagnosis duration. Figure 6 displays changes in pain intensity, sensitivity and feelings towards the limb as well as total device usage time for each participant. Table 3 provides the individual results for each participant based on the quantitative data collected.

**Table 3.** Participant individual results.

Participant	5	6	7	8	11	14	16	18	19	21	Average	Range (min)	Range (max)
Number of days device used (days)	25.0	20.0	30.0	24.0	26.0	29.0	30.0	30.0	29.0	30.0	27.3	20.0	30.0
Days used for 30 min, excluding music player (days)	8.0	20.0	0.0	23.0	0.0	0.0	24.0	21.0	25.0	19.0	14.0	0.0	25.0
Total time used over 30 days, excluding music player (hh:mm:ss)	11:15:00	10:45:00	07:46:39	20:29:00	13:58:00	09:31:06	16:55:00	14:37:14	17:31:00	13:09:19	13:35:44	07:46:39	20:29:00
Days used for 30 min, including music player (days)	8.0	20.0	29.0	23.0	20.0	20.0	28.0	21.0	27.0	29.0	22.5	8.0	29.0
Total time used over 30 days, including music player (hh:mm:ss)	11:15:00	11:01:00	15:47:48	20:30:00	13:58:00	13:29:45	20:05:00	14:37:14	18:30:00	14:44:29	15:19:50	10:21:00	20:30:00
Time 1 – TPD	4.0	2.0	3.0	4.0	4.0	5.0	2.0	3.0	1.0	5.0	3.3	1.0	5.0
Time 2 – TPD	2.0	2.0	4.0	2.0	4.0	3.0	4.0	4.0	1.0	2.0	2.8	1.0	4.0
TPD difference	−2.0	0.0	1.0	−2.0	0.0	−2.0	2.0	1.0	0.0	−3.0	−0.5	−3	2.0
Time 1 – pain interference (T-score)	64.0	68.0	67.9	68.0	66.7	68.0	66.7	63.8	66.7	68.0	66.78	63.8	68.0
Time 2 – pain interference (T-score)	66.7	68.0	67.9	68.0	65.2	66.8	65.5	63.8	65.2	69.4	66.7	63.8	69.4
Pain interference difference (T-score)	2.7	0.0	0.0	0.0	−1.5	−1.2	−1.2	0.0	−1.5	1.4	−0.1	−1.5	2.7
Time 1 – pain intensity	9.0	5.0	8.0	5.0	7.0	8.0	5.0	6.0	8.0	7.0	6.8	5.0	9.0
Time 2 – pain intensity	9.0	7.0	8.0	5.0	6.0	8.0	5.0	7.0	7.0	8.0	7.0	5.0	9.0
Pain intensity difference	0.0	2.0	0.0	0.0	−1.0	0.0	0.0	1.0	−1.0	0.0	0.2	−1.0	2.0
Time 1 – sensitivity	9.0	9.0	8.0	9.0	7.0	10.0	7.0	7.0	10.0	9.0	8.5	7.0	10.0
Time 2 – sensitivity	9.0	9.0	8.0	10.0	8.0	7.0	7.0	3.0	7.0	9.0	8.5	7.0	10.0
Sensitivity difference	0.0	0.0	0.0	1.0	1.0	−3.0	0.0	−4.0	−3.0	0.0	−0.8	−4.0	1.0
Time 1 – feeling towards the limb	8.0	6.0	5.0	9.0	8.0	9.0	8.0	5.0	7.0	7.0	7.2	5.0	9.0
Time 2 – feeling towards the limb	9.0	4.0	5.0	9.0	8.0	8.0	7.0	2.0	7.0	5.0	6.4	2.0	9.0
Feeling towards the limb	1.0	−2.0	0.0	0.0	0.0	−1.0	−1.0	−3.0	0.0	2.0	−0.8	−3.0	−1.0

## Qualitative findings

Ten semi-structured interviews were conducted by JC between February 2022 and February 2023, lasting 19–49 min (mean: 32 min). The median time from device usage end to interview was 4 days. Three descriptive themes were identified.

### Study process and device utilisation

Online questionnaires at T1 and T2 were easy to complete and accepted by participants. The initial video call, and weekly telephone calls with the researcher were helpful. Participants valued talking to someone who understood CRPS. TPD assessments caused some discomfort but were generally straightforward. Participants praised the instructional videos and booklets, with a preference for the video format. The paper diary was straightforward, but participants requested more writing space, a dedicated score section, specific recording instructions, and an electronic version for those with CRPS in their dominant hand.

‘Knowing you were there at the end of the phone or an email if there was a problem, very helpful and very reassuring’ – participant 6

The device initially appeared complex and intimidating to some. A few participants experienced feelings of fear, particularly about wearing the STS near the allodynic area.

‘Once we opened the package and saw everything, it was like oh gosh, I think it's going to be quite complicated. And, then watching the video, it simplified everything’ – participant 5

Most received help from friends or family during setup, though they believed they could manage it themselves at a slower pace. Setup took 30 minutes to an hour. Concentration during the 30-minute device use was initially challenging but improved over time. Participants could split sessions into two 15-minute sessions, but many preferred continuous 30-minute sessions to avoid frequent setup and electrode cleaning.

### Hardware

Participants found the STS bulky, heavy, and intimidating. The most common issue was the stiff, heavy-duty stimulator cable, which was uncomfortable and prone to twisting, and feared to touch the CRPS-affect limb. The cable’s weak connection to the electrode array led to frequent detachment.

‘The actual device I think could be better, eventually with the amount of wires and things like that and the connection’ – participant 19

Other issues included difficulty placing the array in the connector due to unclear orientation indicators and challenges with the hydrogels on the electrodes, requiring frequent replacement. Opinions on the wearable band varied, with some finding the size suitable and others stating the fit was poor.

### Software

Participants generally accepted the games, finding difficulty levels suitable. Game enjoyment was linked to performance, but many, inexperienced in gaming, had limited feedback for improvements. Repetitive and distracting game sounds led some participants to turn them off.

More challenging Blockbuster levels caused initial confusion and frustration but became acceptable with repetition. Suggestions included adding an intermediate level and improving visual clarity.

‘The first two steps are quite easy but that’s good, it gets you started, and I think it eases you into it. Then levels three to five start knocking you about a bit’ – participant 8.

Flower Tower was preferred due to its visual appeal and calming nature, though it lacked motivating prompts. Participants proposed personalising the game with their own garden to enhance engagement.

Music Player received mixed responses; some found it relaxing, others were unsure of its purpose, and some found the stimulation stronger than the games. Slow songs were more popular. Suggestions included adding personal music and diverse images during playback.

Participants suggested software additions, including guided meditation, a game involving falling music notes and expansions on Blockbuster levels.

### Adverse events

Three unrelated adverse events occurred. Four participants reported increased pain, leading to three withdrawals. All four reported symptom reduction after discontinuing device use. No serious adverse events or reactions were reported.

## Discussion

This study assessed the feasibility and practicality of using a novel STS in the home setting for people with long-standing CRPS. Specifically, we wished to establish if the hardware and software were sufficiently engaging and easy to use for a minimum of 30-minutes a day over a 30-day period. Our data demonstrates our device was not successful in achieving this proposed therapeutic dose. We discuss below the study design and device factors that may have contributed to this outcome, and consider lessons learnt, which may assist future medical device trialists, especially for CRPS.

Our study design was informed by Flor and colleagues,^16^ however, the GRADE^43^ quality of this study has been rated as ‘very low’ in a recent systematic review of sensory discrimination training for chronic musculoskeletal pain.^44^ Previous studies of sensory training interventions in CRPS populations, have used different training protocols and sensory discrimination tasks. For example, Moseley et al.^20^ had 11–17 days for the discrimination stage in their four-phase design study in 13 people with CRPS, and the duration of training per day was 24 minutes. This schedule compares well to our data where participants used the training games for a minimum of 30 minutes on an average of 14 ± 10.7 days. Pleger et al.^21^ conducted multidisciplinary ‘sensorimotor treatment protocols’ with patients with CRPS (n = 6), 3 to 4 days a week and in two to three sessions per day which each lasted at least 15 to 30 minutes over a period ranging from 1 to 6 months. Perhaps importantly, the training was first introduced in a face-to-face clinical appointment and delivered by healthcare professionals throughout both studies,^21,22^ supplemented by an additional daily session at home by a trained ‘assistant’ in one study.^21^

As stated above, our study was conducted during the COVID-19 pandemic, which required us to run the study remotely. Our original intention was for participants to attend a face-to-face appointment with the research team, at the start and end of the study. These appointments would have enabled us to capture Time 1 and 2 data in person and provided an opportunity for the participant to be taught how to use the device. The researcher could have watched the participant set up, use, and turn off the device, and supported them with any technical challenges. Potentially, this would have ensured all participants followed the same shutdown process and the 13% data loss experienced by four participants caused by a software implementation issue would have been avoided. Due to requirements for social distancing, this important ‘hands on’ support was replaced with online video conferencing with the researcher, and self-guided instructional videos and documentation. Although the content of the videos and guidance documents were tested by our public contributors, in addition to members of the research team, we believe this lack of in person training at the outset of the study contributed to some of the hardware and software challenges reported by our participants.

Research conducted during and since the COVID-19 pandemic, which has explored the pros and cons of online versus face-to-face teaching, concluded that learning can be successfully achieved in both environments, but a blended approach is considered preferable, especially for practical skills learning.^45,46^ A protocol with a mix of face-to-face and online participant engagement may be beneficial for device development within a clinical trial. There were certainly benefits of delivering a remote study (e.g. reduced resource use for researcher and participant travel, greater geographical reach for recruitment), but for future device studies we would recommend at least one face-to-face appointment to introduce the device to the participant and ensure they are confident and competent to use it as required. Furthermore, we recommend an interim data analysis or access to real time data during the study. This addition to our study would have identified the data that were not being captured when the device was shut down incorrectly.

We asked participants to use our device for a minimum of 30 minutes per day, but we perhaps failed to make it sufficiently clear this related to the time they were engaging in the training games. Participants may have manually timed their 30 minutes from when they first switched on the device. Within this there will have been the time taken to set up and review the stimulation intensity levels. Whilst usage beyond 30 minutes (up to 120 minutes) was encouraged, many may have considered they were meeting the requests of the clinical study despite the logged data being just under 30 minutes. This highlights the importance of PPI in study design, in addition to device design, and pilot testing of all instructional documentation to ensure communication, instructions, and feedback are optimal.

We had worked extensively with CRPS patient contributors since the inception of this device, back in 2008, and in the current study, we consulted with CRPS patient contributors at all stages. However, limitations in funding and time meant we could not do longitudinal, iterative testing of all aspects, and instead we conducted consultations at key stages (e.g. plans for software, hardware, development of recruitment and participant information documents). The UK Standards for Public Involvement stresses the importance of public involvement in research, in particular to ensure impact, the ‘changes, benefits and learning resulting from public involvement are acted on’.^47^ We acted on the feedback we received but the study design, device hardware and software would have potentially benefited from more public contributor involvement, over a longer period of time. Future device studies should include significant resource for PPI and consider the duration of time required for extensive pilot testing of a prototype device.

Pandemic restrictions also required us to rely on participants and a nominated friend or family member, in place of the researcher, to collect TPD data. To the best of our knowledge this approach has not been previously reported, and there are no published validation data for the method we used. In an attempt to reduce measurement errors, we simplified the task by changing the aesthesiometer scale to 5 points from 10 points, but we recognise the risks of potential inaccuracies in data reporting. The use of volunteers for scientific data collection is well established in Citizen Science and reported benefits include: raising awareness of the topic under investigation and scientific endeavour; reduced expenditure for a larger volume of data; and the ability to run a study over a longer time period than may be possible within a traditional academic study.^48^ To ensure data accuracy, appropriate training is required, which we delivered in our study via instructional video. However, verifying data accuracy via an independent assessor would have strengthened our confidence in these data. Our participants stated the instructions were easy to follow and they had no problem with the task, which suggests it may be a viable method to use in future clinical trials, subject to appropriate validation studies.

People with CRPS are known to experience a diverse range of sensory perception changes, in addition to the well described severe pain in their affected limb.^13,15,49^ These altered perceptions include hyperacusis,^50^ a sensitivity to sound. It is of interest that participants reported the sounds within the games were intrusive and they preferred to use the device without the sounds on. All our participants selected the Music Player option over the 30-day period, so sound per se does not appear to have been an issue. A survey of the general population (*n* = 2400) explored game players preferences for sound or no sound when gaming.^51^ Only 8.48% (*n* = 204) of those surveyed used the sound option, the vast majority preferred to play electronic puzzle games without sound. In our device development we had sought views from public contributors about the sounds we included and had thought these may enhance device engagement. Our study findings suggest this may not be the case, and silent devices or sounds with less repetition may be preferable.

Our inclusion and exclusion criteria were tailored to our population of interest (CRPS Type 1), device safety requirements and the restrictions of the COVID-19 pandemic. These meant that some groups of people were excluded from recruitment. For example, those who were not digitally able or had access to a computer and/or telephone to stay in touch with the research team. Future studies, particularly those using e-documentation, should ensure other modes of communication and documentation are available to maximise equity of access to research. We also excluded anyone isolated without a family member or friend to conduct the two-point discrimination assessment. However, this was probably a unique limitation due to the pandemic. As there was no face-to-face component in the study, this did mean we could recruit people from across England, and those who were potentially housebound, which was a strength.

## Strengths and limitations

The strengths of this study were: the collaboration and consultation with people with CRPS during device development; an established multi-disciplinary research team who have worked together in this area for more than a decade; and all data were collected independently of the research team, apart from the final qualitative interviews. A limitation, in addition to those factors already discussed above, was the scale of the funding available to conduct this study. This limited the number of devices we could manufacture and the duration of the study. Our device has not yet proven that we can improve pain, and we found this precluded us from successfully applying for much larger funding awards. Funds were also required to pay external consultants to support our successful application for MHRA approval. Significantly more money would have allowed us to create a more sophisticated and user-friendly device and resourced more time with patient research partners to pre-test our device and provide feedback. We are extremely grateful to our funders, but device development, can be a long, and potentially expensive process. It may be best done with a commercial partner at the outset. However, without proof-of-concept data, finding a commercial partner can also be a challenge.

## Conclusion

This feasibility study, of a novel STS for people with CRPS for use in their home, has described the challenges of creating and testing a robust and professional device within resource constraints and in the context of the COVID-19 pandemic. However, important information about the acceptability and practicality of our device was learnt from participants’ feedback, to help inform why the target duration of device use was not met. For those considering this type of work we recommend:

• working collaboratively with the end user from the outset, and throughout device development, to inform the design and use of the device, and device user manuals;
• securing considerable financial resource to cover MHRA costs, development of hardware and software, and payment of patient research partners in addition to costs for the conduct of the study. Working with a commercial partner from the outset maybe optimal;
• having at least one face-to-face consultation at the start of the study to ensure correct device set-up and use;
• conducting interim data analysis on device captured data, or have access to real time data during the study so early ‘trouble-shooting’ can be conducted;
• being mindful of the generalised heightened sensitivity in people with CRPS, which may influence their tolerance of any device sounds;
• using instructional videos and e-study outcome measures and consent, as these were well received by our participants.

## Supplemental Material

Supplemental Material - Feasibility study of a home-based sensory training system (STS) device for type 1 complex regional pain syndrome in England: Lessons learnt Supplemental Material for Feasibility study of a home-based sensory training system (STS) device for type 1 complex regional pain syndrome in England: Lessons learnt by Jessica Coggins, Sharon Grieve, Lisa Buckle, Darren Hart, Alison Llewellyn, Mark Palmer, Moniek Wittens, and Candida McCabe in British Journal of Pain

## Data Availability

The quantitative dataset will be available via the Figshare repository platform. The transcripts from the qualitative interviews are not available to share due to concerns around anonymity.*
