# Supplementary material for: Feasibility study of a home-based sensory training system (STS) device for type 1 complex regional pain syndrome in England: Lessons learnt
Source: Br J Pain. 2025 Aug 29;19(6):417–33. doi: 10.1177/20494637251371592 (PMC12397103; doi:10.1177/20494637251371592)
Supplement: Supplemental Material - Feasibility study of a home-based sensory training system (STS) device for type 1 complex regional pain syndrome in England: Lessons learnt [file sj-pdf-1-bjp-10.1177_20494637251371592.pdf]

Feasibility study of a home-based Sensory Training System (STS) Device for Type 1 Complex Regional Pain Syndrome in England: lessons learnt.

Supplementary file: participant demographics

**Participant ID**	**Gender**	**Age at time 1**	**CRPS diagnosis time (years)**	**Affected limb**	**Participation status**	**Days device used before withdrawal**
4	Female	70	4.8	Right arm/hand	Withdrew – Increased symptoms/pain	3
5	Female	63	5.2	Left leg/foot	Completed	
6	Female	62	4.3	Right leg/foot	Completed	
7	Female	24	5.1	Right arm/hand	Completed	
8	Male	61	4.3	Right leg/foot	Completed	
11	Female	78	26.5	Left arm/hand	Completed	
12	Female	74	14.0	Left leg/foot	Withdrew – Increased symptoms/pain	3
13	Female	47	8.4	Right arm/hand	Withdrew – Increased symptoms/pain	10
14	Male	61	10.5	Right leg/foot	Completed	
16	Female	68	8.5	Left leg/foot	Completed	
15	Female	44	11.0	Right leg/foot	Loss to follow up	Unknown
18	Male	71	12.6	Right leg/foot	Completed	
19	Female	51	8.6	Right leg/foot	Completed	
21	Female	25	8.2	Right leg/foot	Completed	
23	Male	54	4.1	Left arm/hand	Withdrew – personal reasons not related to the study	Unknown
